# A Retrospective Epidemiological Study of the Incidence and Risk Factors of Salmonellosis in Bahrain in Children during 2012–2016

**DOI:** 10.3390/pathogens8020051

**Published:** 2019-04-17

**Authors:** Simone Perna, Zahraa Alaali, Tariq A. Alalwan, Essam Moahmmed Janahi, Sakina Mustafa, Mariangela Rondanelli, Ali Salman Bin Thani

**Affiliations:** 1 Department of Biology, College of Science, University of Bahrain, Sakhir Campus, Sakhir 976, Bahrain; zahraaj.alaali@gmail.com (Z.A.); talalwan@uob.edu.bh (T.A.A.); emohammed@uob.edu.bh (E.M.J.); sakina117@hotmail.com (S.M.); abinthani@uob.edu.bh (A.S.B.T.); 2 IRCCS Mondino Foundation, 27100 Pavia, Italy; mariangela.rondanelli@unipv.it; 3 Department of Public Health, Experimental and Forensic Medicine, Unit of Human and Clinical Nutrition, University of Pavia, 27100 Pavia, Italy

**Keywords:** salmonellosis surveillance, Bahrain, children, pediatric, epidemiology, food- and water-borne pathogen, incidence rates

## Abstract

Salmonellosis is one of the major public health concerns in Bahrain as it has increased rapidly during the past few years. This study aims to determine the prevalence of salmonellosis in children and the possible risk factors such as age, geographical area, nationality, gender, unsafe drinking water, infant born weight and gastrointestinal disease. The cases of salmonellosis in children reported by the Ministry of Health of Bahrain ranged from 21 to 26 per 100,000 population during the period 2012–2016. Salmonellosis cases were geographically concentrated in the capital and northern regions. Statistical analysis showed a significant difference in the number of salmonellosis cases between Bahrainis and non-Bahrainis based on region, and gender (*p* < 0.001). In the Bahraini cohort, there was an association between the increase of cases and the number of gastrointestinal disease-related deaths (*p* < 0.05). In addition, unsafe water (over the level of 2.14%) and low-birth weight (<3.100 g) were associated, but not statistically significant (*p* = 0.086 and *p* = 0.126, respectively) with the increase of salmonellosis cases. Despite the decline in the number of cases, the results of this study contribute to the understanding of the epidemiology of *Salmonella* in humans and this, in turn, will help develop and implement preventative measures.

## 1. Introduction

*Salmonella* is one of the top bacterial pathogens to cause illnesses, hospitalizations and deaths annually. Centers for disease control and prevention (CDC) estimate about 23,000 hospitalizations in the United States and 155,000 of deaths per year globally due to salmonellosis diseases specifically gastroenteritis disease [1]. To date, over 2600 *Salmonella* serotypes were identified and the majority of these serotypes cause human infections. Infected individuals with *Salmonella* develop diarrhea, fever and abdominal cramps after 12 to 72 h of infection. The illness usually lasts for 10 days or less [2].

Considering that *Salmonella* species are pathogenic, it comes to no surprise that they are the top food- and waterborne bacterial pathogens responsible for food spoilage and a variety of infections in humans. The ability of *Salmonella* to be transmitted through a fecal-oral route of infection makes it one of the major worldwide public health concerns [3]. Nowadays, *Salmonella* outbreaks mainly occur among vulnerable populations such as infants and young children due to the consumption of powdered infant formula. Powdered infant formula manufacturing is one of the most high-risk types of food manufacturing [2]. The risk of contamination of the manufacture environments may occur as a result of poor operational practices, inadequate hygiene, improper maintenance, substandard facility and equipment design [1]. Also, the inadequate ingredient control is one of the risk factors for *Salmonella* contamination. The ability of *Salmonella* to survive and remain stable for a long time in processed and dried (low water activity of 0.2 at 22 °C) products like powdered infant milk has been previously reported [4]. Moreover, infants are more likely to experience *Salmonella* infections due to their less well-developed immune system and their lack of competing intestinal microflora [5].

The increase in bacterial diseases worldwide is accounted for to a large extent by a parallel global prevalence of multidrug resistant bacteria, such as extended-spectrum beta-lactamases (ESBLs) pathogens. These pathogens including *Salmonella* are associated with high rates of mortality and morbidity, which poses a serious threat to the health sectors in many countries. A rapid continuous increase in bacterial diseases has led to the increase in numbers of death among the different age groups in Bahrain [6]. Among 11,886 Enterobacteriaceae species isolated from hospitals in Bahrain during 2005–2006, 2695 (22.6%) were ESBL producers.

Overall, the prevalence of bacterial diseases including salmonellosis was noticeable worldwide mainly in infants and children below the age of five years. According to the World Health Organization (WHO) data [5], 550 million people fall ill each year, including 220 million children under the age of five years from diarrheal diseases, with *Salmonella* being one of the four major causes of diarrheal diseases globally. This has prompted the global health community to alert the public about this disease. For instance, the Ministry of Health in Bahrain has taken a number of initiatives to educate the public about salmonellosis disease in order to prevent it and reduce its ratio in terms of cases to the total population. In this regard, the Ministry of Health in Bahrain started documenting the number of cases in each hospital and health centers. According to the records and statistics data, there was an increase in salmonellosis within the past few years specifically from 2012 to 2015 where it reached its highest number of cases (288 cases to 336 cases, respectively) [6].

The objectives of this study were to (1) determine whether there was any association between *Salmonella* infections and geographical region, (2) detect any possible correlation of *Salmonella* infections with ethnic groups in the Kingdom of Bahrain, (3) determine whether water and birth weight were possible factors for salmonellosis and (4) detect if a relationship existed between gastrointestinal disease-related deaths and salmonellosis.

## 2. Methods

### 2.1. Ethical Issues

The study was approved by the Ministry of Health Research Committees reviews.

This decision was made as the obtained data were completely anonymized with no possibility of being linked directly or indirectly to human subjects.

### 2.2. Data Source

Salmaniya Medical Complex is the main public hospital and the only medical complex in the Kingdom of Bahrain that was established in 1957. The cases of salmonellosis were recorded in this hospital as well as in other private hospitals via laboratory examinations. Biochemical tests were done to confirm the diagnosis by isolating *Salmonella* from clinical specimens (stool or blood). The specimens were plated on several selective agar media (MacConkey agar, eosin-methylene blue) as well as into an enrichment broth such as tetrathionate. The biochemical reactions of suspicious colonies were then determined on triple sugar iron agar and lysine-iron agar, and a presumptive identification was made. The biochemical identification of *Salmonella* was achieved using the matrix-assisted laser desorption ionization-time of flight (MALDI-TOF) mass spectrometry (MS) that enabled the rapid testing of 10–20 different biochemical parameters simultaneously.

Recorded cases included patients (with information such as age, gender and location) who attended hospitals in Bahrain during 2012–2016. The statistical data was obtained from the Ministry of Health, Bahrain, and is the only data regarding salmonellosis cases in Bahrain. The total population in Bahrain during 2012–2016 was based on the published statistics of the Bahrain Central Informatics Organization.

We analyzed data from 2012–2016 for the age group 0–5 years old and for both genders. In addition, we analyzed the data for all regions (Capital, Muharraq, Northern and Southern governorates). However, the central region was excluded from further analysis because of the lack of data due to its merger with other governorates.

### 2.3. Statistical Analysis

Statistical analysis was performed using the SPSS version 21 (IBM).12 Descriptive statistics (N, % and mean ± standard deviation (SD) were calculated to assess the number of cases, nationalities, gender, and serotype. Incidence rates (IR) of salmonellosis cases were calculated per 100,000 population per year. The independent t test was applied to test the difference between prevalence rates compared to the category of water (safe versus unsafe) and birth weight (under weight versus normal weight).

## 3. Results

### 3.1. General Data

As reported in (Table 1), the health statistical data for years from 2012 to 2016 were available on the Ministry of Health website, which were used for the purpose of this study. The total number of cases of salmonellosis in the different governorate regions in Bahrain were 288 cases in 2012, 277 cases in 2013, 331 cases in 2014, 336 cases in 2015 and 300 cases in 2016.

Based on the total population and in different years, the salmonellosis cases were determined for 100,000 persons in Bahrain. The highest number of cases reported was in 2014, with 26.675 cases followed by 24.492 cases per 100,000 in 2015. In 2016, there was a decline in the number of salmonellosis cases (21.050), representing the lowest number of cases reported since 2014 (Table 1).

### 3.2. Sum of Salmonellosis Cases in Bahrain

The total number of cases reported during 2012–2016 in the five different governorates/regions showed that the highest cases of salmonellosis occurred in the Capital region with total cases of 560, followed by the Northern region with 440 cases and Muharraq with 219 cases, whereas the lowest cases occurred in the Southern governorate with 186 cases and the Central region with 127 cases (Table 2). However, the Central governorate merged with the other regions in 2014, resulting in the lack of recorded data for this region specifically.

### 3.3. Changes in Salmonellosis Mean Cases

**Figure 1:**
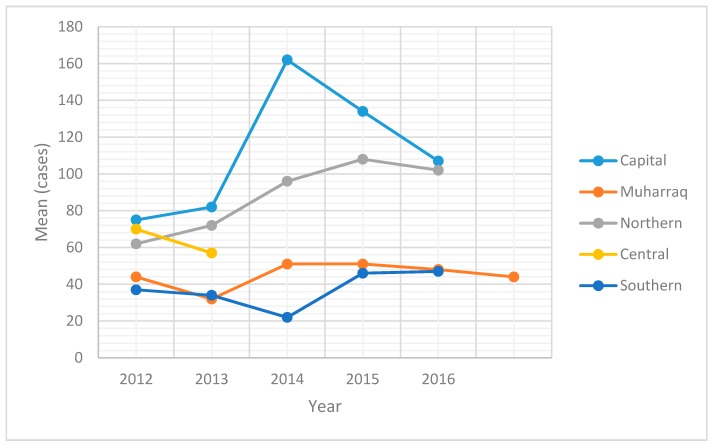
Mean cases of salmonellosis in children for the different governorates/regions in 2012–2016.

Statistical analysis for the mean cases was done to observe the changes in salmonellosis cases in the Capital, Central, Muharraq, Northern and Southern regions from 2012–2016. The mean cases per 100,000 population increased in the Capital region from 75 in 2012 to 82 and 162 in 2013 and 2014, respectively. However, it decreased to 134 and 107 in 2015 and 2016, respectively as shown in (Figure 1). For the Northern region, the mean cases per 100,000 population increased from 62 in 2012 to 108 in 2015 and again decreased to 102 in 2016. As for Muharraq and the Southern governates, the mean cases were not stable, and changes occurred in them frequently.

The statistical analysis results for these data showed that the highest mean was in the Capital region with 32% and the lowest mean was in the Southern region with 11% (Figure 1). Also, the statistical analysis indicated that there was a significant difference (*p* < 0.001) between the regions during the period from 2012–2016.

### 3.4. Salmonellosis Relationship with Ethnic Groups and Gender

To determine the correlation of salmonellosis with ethnic groups, the mean of cases was analyzed for both Bahrainis and non-Bahrainis in each region. This analysis showed that the highest mean cases for Bahrainis were in the Capital region whereas the highest mean cases for non-Bahrainis were in the Northern region. Moreover, the analysis confirmed that there was a significant difference for the percentage mean cases in all regions (Table 3).

By comparison with the mean cases between Bahraini and non-Bahraini males in each region, there was a significant difference in all of the regions except the Muharraq governate due to its P value (0.072). Moreover, mean cases for Bahraini males was at its highest in the Capital region and for non-Bahraini males was in the Northern region (Table 3).

Similarly to the mean cases of non-Bahraini males, the mean cases for non-Bahraini females were at the highest in the Northern region. The statistical analysis also proved that the differences in mean cases between Bahraini and non-Bahraini females in the Northern, Muharraq and Southern regions were significant (*p* < 0.01) as shown in (Table 3).

The mean cases for Bahraini males (0.05%) were almost close to that of females (0.06%) in the same region (Southern region). In addition, there was a significant difference (*p* < 0.01) in this region and all other regions (Table 3).

According to the statistical analysis (Table 3), there was a statistical difference of the mean cases for non-Bahraini males and females (*p* < 0.01). In the Northern region, the mean case for non-Bahraini males was (0.13) and females (0.30), which represents the highest among the other regions.

**Figure 2:**
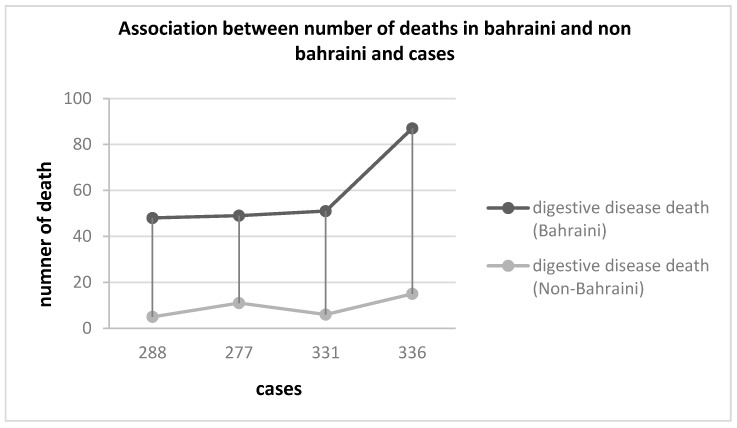
Association between number of deaths of Bahraini and non-Bahraini and cases of salmonellosis.

There was an association between the number of deaths due to the digestive disease and salmonellosis cases in which the statistical analysis showed that as the salmonellosis cases increased, the number of deaths specifically among Bahrainis increased. Also, Bahrainis were more likely to experience higher mortality rates than non-Bahrainis with the increase in salmonellosis cases (Figure 2).

### 3.5. Salmonellosis and Unsafe Water

**Figure 3:**
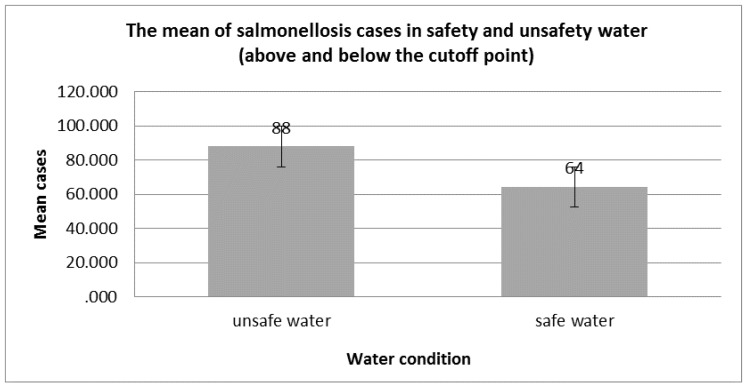
The mean of salmonellosis cases in safety and unsafety water (above and below the cutoff point).

As a result of the descriptive analysis, the cutoff point of 2.14 was considered to separate the potable water from the unsafe water (following the 50th percentile of overall samples). The mean number of cases from unsafe water was 88.00 while the mean number for safe water was 64.24 cases, which confirms the positive correlation between unsafe water and the increase of salmonellosis cases (Figure 3). However, the statistical analysis showed that *p* = 0.086 for mean salmonellosis cases is almost near (*p* < 0.05) but there were no significant differences.

### 3.6. Birth Weight Category and Number of Cases

**Figure 4:**
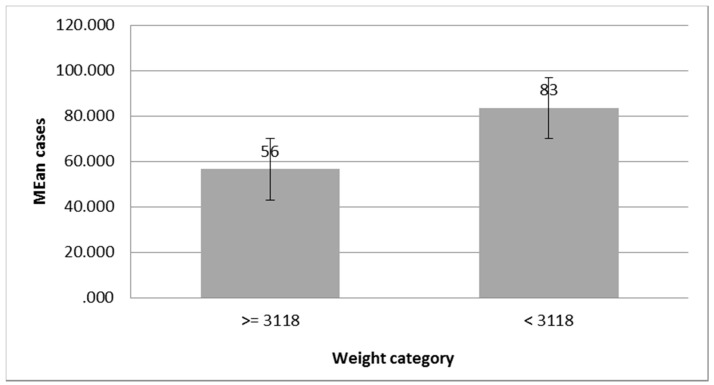
Mean cases of salmonellosis and weight in birth (above and below the cutoff point).

The number of cases of salmonellosis increased when the weight in birth was below 3118 g (cutoff point) and it decreased when the weight was 3118 g or more. The number of cases of newborns with a weight of less than 3118 g reached 83.38 cases. On the other hand, the number of cases in newborns with weight above the cutoff point was 56.50 cases (Figure 4). Moreover, there was no significant difference in the number of cases (*p* = 0.129).

### 3.7. Study Strengths and Limitations

This study has a number of strengths and weaknesses. Strengths include that all of the data were taken directly from the Public Health Directorate at Bahrain Ministry of Health, which is the main source for data from all hospitals in Bahrain. Also, the study focused on analyzing the cases of salmonellosis in the most infected group (children below five years in age).

A limitation of the study was that it only covered five years, which is considered as a short period of time. Another limitation is the absence of antibiotic resistance data related to *Salmonella* species that might be one of the risk factors to increase the number of cases and deaths.

## 4. Discussion

This study, the first of its kind in Bahrain, reports on the cases of salmonellosis in Bahrain, specifically in children below five years of age. The results showed that children between 0–5 years old were susceptible to salmonellosis ranging from 21.050 to 26.675 cases in 100,000 persons. Data on possible risk factors was evaluated as the second outcome of this present study. One possible risk factor was the contamination of the water supply with *Salmonella* and other bacterial pathogens (representing 2.14% of the total sample analyzed), in which the mean number of cases from unsafe water reached 88. This is not completely surprising given that Bahrain depends principally on groundwater extraction to meet its irrigation and domestic needs. Moreover, a study on the contamination of potable water in Al-Ahsa region of neighboring Saudi Arabia found that the underground water supplied to the consumers via water vehicles was not suitable for drinking [7]. For this reason, further studies and investigation on the potability of water supplies in Bahrain are recommended.

The study also identified low birth weight as a predictor of risk for salmonellosis. In a previous study by Hamadeh et al. [8] to determine the incidence rate of foodborne climate-related disease and their seasonal variation, the authors found that a relationship existed between *Salmonella* cases and climate change. However, their study did not consider salmonellosis and no other possible risk factors were identified.

In a recent similar epidemiological study carried out in the neighboring Gulf country of Qatar, it was reported that the incidence of non-typhoidal salmonellosis decreased during the study period of 2004 to 2016 [9]. The authors attributed this to the improvement in the food chain and sanitation in the country. Similarly, there was a decline in salmonellosis cases in Bahrain from 331 cases to 300 cases during the study period (2012–2016) due to the improvement in food hygiene standards and surveillance system. For instance, an international outbreak of *Salmonella* was linked to a contaminated shipment of sprouted chia seed powder that was distributed globally including Bahrain in 2013–2014. The food recall warnings issued by the local health authorities ensured the removal of the contaminated sprouted chia seed powder products from the marketplace [10]. Another example was the successful recall and disposal of *Salmonella*-contaminated milk that was imported to Bahrain from France [11]. Despite the decline in the number of salmonellosis cases in Bahrain as a result of the implementation of international standards for food safety and hygiene and the maintenance of a surveillance system for imported foods, the incidence rate in 2012 was high at 23.21 per 100,000 persons. Lower rates were reported in Qatar with 18.1 cases per 100,000 habitants [9].

One of the novel approaches to identify the epidemiological characteristics of *Salmonella* was demonstrated by Boore et al. [12]. In their study, the authors reported that the number of salmonellosis cases in the United States was 15 per 100,000 persons. In comparison, the number of cases was higher in Bahrain with 26.68 cases per 100,000 inhabitants. The high rate of salmonellosis cases in Bahrain may be attributed to the wide distribution of *Salmonella*-contaminated foods. In fact, a salmonellosis outbreak was associated with a local restaurant in 2014 [11]. Another possible explanation could be the omnipresent source like water as our study results indicated. It must be mentioned, however, that the weakness regarding this data is related to a lack in terms of water contamination microorganisms in overall samples of unsafety water.

In terms of geographical location, our study showed that salmonellosis cases were high in both the Capital (32%) and Northern (26%) regions, however they were in the same low range in the Muharraq (13%), Southern (11%) and Central (18%) regions. This is in agreement with the study of Boore et al. [12] which reported an approximately equal low number of salmonellosis cases in—several US states while other states had a higher number of cases. Moreover, the similar rate of salmonellosis cases in the different regions may suggest a homogenous source or a relatively even geographic distribution source such as nationally distributed food products. In contrast, the higher cases of salmonellosis in specific regions could be due to the natural restricted reservoir in specific local habits or food products.

On the other hand, the same US study [12] reported similar findings to our study with regard to the higher number of salmonellosis cases observed in females compared to males. The possible exposure to fresh produce and the food handling practices might explain these results. In addition, the variation in traditional food exposures, including the home-prepared foods sold on the street by unlicensed Bahraini retailers could explain the significant differences in salmonellosis cases and digestive disease-related deaths between Bahrainis and non-Bahrainis.

Furthermore, the highest salmonellosis cases in our study occurred in children below five years of age, which was similar to that of Boore et al. [12]. This is probably due to the fact that this age group is more likely to seek healthcare, thereby resulting in lower numbers of unreported cases in comparison to the other age groups. Adding to that, the probable link between low-birth weight and immunocompromised infants and the increased incidence of salmonellosis cases [13].

This study is a basic fundamental for the Bahrain Vision of 2030. Bahrain will be a leading centre for modern medicine, offering high-quality and financially sustainable health care in the region.

Salmonellosis remains an important human disease in Bahrain and presents many challenges to the food and agriculture industries and those charged with the protection of public health. Infection rates will only be reduced if there is the closest possible working relationship between all those involved with food production and the government agencies with responsibility for food safety.

In conclusion, this study identifies unsafe water supplies, low-birth weight, nationality, gender, age, geographical region and gastrointestinal diseases as possible major risk factors to salmonellosis infections, either directly or indirectly. Nevertheless, further clinical and experimental studies are needed to fully explore other possible risk factors of salmonellosis in Bahrain.

## Figures and Tables

**Table 1:** Cases of salmonellosis per 100,000 persons in relation to total population and years.

Year	Total Population in Bahrain	Cases in Children	Cases per 100,000 Persons
2016	1,425,171	300	21.050
2015	1,371,855	336	24.492
2014	1,240,862	331	26.675
2013	1,240,862	277	22.323
2012	1,240,862	288	23.210

**Table 2:** Total cases of salmonellosis for the years 2012–2016 in different governorates/region.

Governorates/Region	Capital	Central	Muharraq	Northern	Southern
Total cases of salmonellosis in children	560	127	219	440	186
Cases per 100,000 persons	21.750	-	19.990	27.765	14.135

**Table 3:** Mean cases of salmonellosis for Bahraini and non-Bahraini males and females in each region.

Region	Mean Cases for Males (%)		Sig.	Mean Cases for Females (%)		Sig.
Capital	Bahraini	0.13	*p* < 0.001	Bahraini	0.13	*p* = 0.47
	Non-Bahraini	0.04		Non-Bahraini	0.12	
Muharraq	Bahraini	0.07	*p* = 0.07	Bahraini	0.07	*p* < 0.001
	Non-Bahraini	0.06		Non-Bahraini	0.13	
Northern	Bahraini	0.07	*p* < 0.001	Bahraini	0.07	*p* < 0.001
	Non-Bahraini	0.13		Non-Bahraini	0.30	
Southern	Bahraini	0.05	*p* < 0.001	Bahraini	0.06	*p* < 0.001
	Non-Bahraini	0.03		Non-Bahraini	0.09	
Region	Mean cases for Bahraini (%)		Sig.	Mean cases for Non-Bahraini (%)		Sig.
Capital	Males	0.13	*p* < 0.01	Males	0.04	*p* < 0.01
	Females	0.13		Females	0.12	
Muharraq	Males	0.07	*p* < 0.01	Males	0.06	*p* < 0.01
	Females	0.07		Females	0.13	
Northern	Males	0.07	*p* < 0.01	Males	0.13	*p* < 0.01
	Females	0.07		Females	0.30	
Southern	Males	0.05	*p* < 0.01	Males	0.03	*p* < 0.01
	Females	0.06		Females	0.09	
